# Research progress on the effect of gut and tumor microbiota on antitumor efficacy and adverse effects of chemotherapy drugs

**DOI:** 10.3389/fmicb.2022.899111

**Published:** 2022-09-23

**Authors:** Beibei Yin, Xuan Wang, Fang Yuan, Yan Li, Ping Lu

**Affiliations:** ^1^Department of Oncology, The First Affiliated Hospital of Shandong First Medical University & Shandong Provincial Qianfoshan Hospital, Shandong Key Laboratory of Rheumatic Disease and Translational Medicine, Shandong Lung Cancer Institute, Jinan, China; ^2^Department of Digestive Endoscopy, The Affiliated Hospital of Shandong University of TCM, Jinan, China; ^3^Department of Cardiovascular Surgery, Shandong Engineering Research Center for Health Transplant and Material, The First Affiliated Hospital of Shandong First Medical University & Shandong Provincial Qianfoshan Hospital, Jinan, China

**Keywords:** chemotherapy, gut microbiota, tumor microbiota, microbiome, antitumor efficacy

## Abstract

Chemotherapy is one of the most effective methods of systemic cancer treatment. Chemotherapy drugs are delivered through the blood circulation system, and they can act at all stages of the cell cycle, and can target DNA, topoisomerase, or tubulin to prevent the growth and proliferation of cancer cells. However, due to the lack of specific targets for chemotherapeutic agents, there are still unavoidable complications of cytotoxic effects. The effect of the microbiome on human health is clear. There is growing evidence of the potential relationship between the microbiome and the efficacy of cancer therapy. Gut microbiota can regulate the metabolism of drugs in several ways. The presence of bacteria in the tumor environment can also affect the response to cancer therapy by altering the chemical structure of chemotherapeutic agents and affecting their activity and local concentration. However, the underlying mechanisms by which the gut and tumor microbiota affect cancer therapeutic response are unclear. This review provides an overview of the effects of gut and tumor microbiota on the efficacy and adverse effects of chemotherapy in cancer patients, thus facilitating personalized treatment strategies for cancer patients.

## Introduction

Trillions of microbes are present in the human gut. Although the gut microbiota maintains a relatively stable composition throughout the human lifetime, the ratio of different bacteria is affected by the gut microecosystem and changes in the gut microbiota have profound effects on the host (Panebianco et al., 2018a; Liu et al., 2019). Gut microorganisms are involved in a variety of physiological events, including the provision of nutrients and vitamins, the metabolism of drugs and toxicants, the protection of the host from pathogens, the development of the immune system and the maintenance of epithelial mucosal homeostasis, and are important determinants of the physiological or pathological status of the host (Ding et al., 2018; Whisner and Athena Aktipis, 2019; Wong et al., 2019).

Cancer is a globally challenging health problem of human. Despite many advances in cancer treatment, heterogeneous responses and resistance to chemotherapeutic agents remain challenges for cancer treatment. The chemotherapeutic agents lack specific targets, and therefore specific markers and methods for predicting therapeutic efficacy are still lacking.

Since the 1960s, drug metabolism by gut microbes has been widely recognized (Scheline, 1968). The relationship between gut microbiota and malignant tumor therapy has attracted much attention in current tumor research and treatment. Currently, there are several animal studies confirming that complex interactions do exist between the chemotherapeutic drugs and the microbiome. For example, it has been reported that gut microbiota may positively or negatively regulate the activity of some chemotherapeutic drugs, including through enzyme biotransformation, thus altering the efficacy of chemotherapeutic drugs (Lehouritis et al., 2015). Numerous evidence suggests that inflammation and tumor microenvironment may play an important role in promoting chemotherapy resistance. Emerging evidence suggests an interesting relationship between gut microbiota and chemotherapy resistance (Deng et al., 2018; Liu et al., 2019). Antitumor drugs also have toxic effects on normal cells, which may even lead to the occurrence of life-threatening adverse reactions. Therefore, it is of great importance for tumor treatment to achieve the maximum therapeutic effect while maintain the minimum toxic side effects. In recent years, there has been increasing evidence that gut bacteria are closely related to the pharmacological effects of chemotherapeutic drugs [such as Fluorouracil (5-Fu), Cyclophosphamide, Irinotecan, Oxaliplatin, and Gemcitabine], and gut microbiota can affect the antitumor effects of chemotherapeutic drugs by altering drug bioavailability and drug re-metabolism. Therefore, the regulation of gut microbiota is expected to be a new way to improve therapeutic efficacy and reduce the toxicity of chemotherapeutic drugs. Herein, the effects of gut microbiota and tumor microbiota on the antitumor efficacy and adverse effects of chemotherapeutic drugs were summarized (Table 1).

**Table 1:** The effect of gut and tumor microbiota on the antitumor efficacy and adverse reaction of chemotherapeutics.

Chemotherapeutics	Effect of gut microbiota on the antitumor efficacy of chemotherapeutics	Effect of gut microbiota on the adverse reaction of chemotherapeutics
Cyclophosphamide	Ectopic gut microbiota induces tumor cell death and enhances the therapeutic effect of cyclophosphamide Wilkinson et al. (2018)	–
5-Fluorouracil (5-Fu)	The abundance of *Enterobacteriaceae* (facultative Gram-negative bacteria) is increased after 5-Fu treatment Takemura et al. (2014) *Fusobacterium nucleatum* can promote 5-Fu resistance by increasing the expression of BIRC3 in colorectal cancer Zhang et al. (2019)	5-Fu causes imbalance of gut microbiota, which may lead to gut mucositis, bacteremia or sepsis. Antibiotic cocktail therapy reduces the antitumor efficacy of 5-Fu in mice, but probiotics supplementation after 5-Fu treatment does not significantly increase the efficacy of 5-Fu treatment Yuan et al. (2018)
Gemcitabine	cdd wild-type *Escherichia coli* is associated with gemcitabine resistance; *γ-amastigotes* can induce gemcitabine drug resistance Choy et al. (2018) The combined use of antibiotics can enhance the activity of gemcitabine	–
Platinum	Gut microorganisms promote the ROS secretion and increase the efficacy of platinum; Anti-gram positive antibiotics are associated with decreased anticancer effect of platinum salts in animal models	Gut microbiota involves in regulating other common side effects of cisplatin, such as ototoxicity, mucositis and weight loss. Oral D-methionine has protective effect on mucositis induced by cisplatin Hamstra et al. (2018)
Irinotecan	–	Combination of irinotecan and selective bacteria β-glucuronidase inhibitors can prevent colon injury or diarrhea Gui et al. (2015) The use of probiotics can reduce the incidence of diarrhea caused by irinotecan Roy and Trinchieri (2017)

## Effect of gut and tumor microbiota on the antitumor efficacy of chemotherapeutic drugs

### Cyclophosphamide

Cyclophosphamide is a cell cycle non-specific alkylating agent that acts mainly on the S-phase and exerts cytotoxic effects by affecting DNA synthesis. It is commonly used for the treatment of lymphoma, leukemia, neuroblastoma, and retinoblastoma, as well as ovarian, breast, endometrial, and lung cancers. However, the development of chemotherapy resistance seriously limits its efficacy (Kroemer et al., 2013).

In recent years, it has been shown that gut microbiota is involved in the regulation of the host immune response triggered by Cyclophosphamide. Studies in mouse models have found that Cyclophosphamide could disrupt the intestinal mucus layer, and alter gut microbiota, which was accompanied with the translocation of specific Gram-positive bacteria into secondary lymphoid organs. Cyclophosphamide can disrupt the mucus layer of the gut and change the gut microbiota, leading to accumulation of monocytes in the lamina propria and translocation of Gram-positive bacteria in mesenteric-positive lymph nodes and spleen (Daillère et al., 2016a). Cyclophosphamide increases the permeability of the gut mucosa, which allows the gut microbiota to translocate from the intestine to the mesenteric lymph nodes (Viaud et al., 2013). These ectopic bacteria can promote the differentiation of CD4-positive T cells to Th-17 memory T cells and promote the secretion of IL-17 and IFN (interferon)-γ, thus inducing immunogenic tumor cell death (Daillère et al., 2016a). Besides, it is found that the Th-17 cellular responses and the antitumor effects of Cyclophosphamide were reduced in antibiotic-treated germ-free mice without Gram-positive bacteria (Iida et al., 2013). The antitumor effect of Cyclophosphamide was restored in colony-deficient mice after *in vitro* establishment and proliferation of Th-17 cells by relay transfer (Viaud et al., 2013), suggesting that gut microbiota can further enhance the antitumor efficacy of Cyclophosphamide by modulating the immune response. However, the specific microbiota involved in this process is not clear.

Viaud et al. found that Cyclophosphamide increased the abundance of Gram-positive bacteria such as *Lactobacillus johnsonii*, *Lactobacillus rhamnosus*, and *Enterococcus hirae* in the intestines of mice (Daillère et al., 2016b). The antitumor effect of Cyclophosphamide is attenuated in mice treated with antibiotics. In non-metastatic sarcoma mouse models, specific Gram-positive bacteria (*Lactobacillus lactis*, *Enterococcus*, and filamentous segmented bacteria) are essential for mediating the antitumor response to Cyclophosphamide. Gram-negative *Barney’s bacteria* are found to affect the antitumor effects of Cyclophosphamide by increasing the infiltration of T cells in cancerous lesions (Daillère et al., 2016a; Rea et al., 2018; Wilkinson et al., 2018).

### Methotrexate

Methotrexate is an anti-folate drug, and can inhibit dihydrofolate reductase, thus preventing the DNA synthesis of tumor cells, inhibiting the growth and proliferation of tumor cells and exerting antitumor effects (Djerassi, 1967; Bertino, 1979). It is used clinically for the treatment of acute leukemia, especially acute lymphoblastic leukemia, choriocapillaris epithelial carcinoma, staphyloma, etc., with good therapeutic efficacy. It is also effective for head and neck tumors, breast cancer, lung cancer, and pelvic tumors. Fijlstra et al. determined the structural characteristics of jejunal tissue and gut bacteria in a rat model of gastrointestinal mucositis induced by Methotrexate (Fijlstra et al., 2015). They found that the level of gut inflammation was increased, and the length of villi was decreased after Methotrexate treatment, compared to the control group (Fijlstra et al., 2015). The relative abundance of most genera in the gut microbiota of rats treated with Methotrexate decreased, which was associated with diarrhea and shortened villus length; the relative abundance of Streptococcus decreased; but, the relative abundance of *Bacteroides* increased.

### 5-Fu

5-Fu is converted intracellularly to Fluorouracil deoxynucleotides, which can inhibit DNA synthesis by inhibiting thymine nucleotide synthase. 5-Fu is widely used for the treatment of colorectal cancer (Lee et al., 2016; Mcquade et al., 2017). However, currently, only 10 to 15% of patients with advanced colorectal cancer have a positive response to 5-Fu therapy (González-Vallinas et al., 2013; Lee et al., 2014; Ribeiro et al., 2016). Reducing drug resistance and improving tumor response rates have become key issues of tumor treatment.

Several animal studies have shown (Takemura et al., 2014) that the abundance of Enterobacteriaceae (parthenogenic Gram-negative bacteria) increased after 5-Fu treatment and that mice receiving 5-Fu chemotherapy exhibited ecological dysregulation of gut microbiota (Longley et al., 2003). Other study has shown that 5-Fu treatment resulted in a significantly higher relative abundance of *Microtrichophyceae*, *Bacteroides*, *Odorobacteria*, *Mu-cispirillum*, and *Blauti* in the gut than controls and induced significant changes in biodiversity and community composition (Yuan et al., 2018).

Scholars in China have demonstrated that *Fusobacterium nucleatum* could promote 5-Fu resistance by upregulating BIRC3 expression in colorectal cancer (Zhang et al., 2019). The use of antimicrobial drugs disrupted gut microbiota, thus reducing the therapeutic effect of 5-Fu against colorectal cancer in mice. However, supplementation with probiotics after 5-Fu treatment did not significantly improve the therapeutic effect. Other studies have found that 5-Fu can significantly reduce the number of actinomycetes and alter the abundance of *Enterobacter Hormaeche* and *Edwards*, *Lachnospiraceae*, *Escherichia coli*, *Bacteroidaceae*, and *Lactobacillus*. In contrast to the results of previous studies (Justino et al., 2014; Li et al., 2017), all these studies suggest that gut microbiota exert an important effect on the anti-tumor efficacy of 5-Fu. A systematic study on the response mechanism of gut microbiota to 5-Fu using *Caenorhabditis elegans* revealed that bacteria regulated the host response to chemotherapeutic drugs through an active metabolic mechanism (García-González et al., 2017). These studies relied on targeted PCR or culture methods and could not assess the impact of chemotherapy on the broad-spectrum gut microbiota. Therefore, in 5-Fu therapy for tumor, it is a challenge to use probiotics for treating gut ecological dysbiosis.

### Gemcitabine

Gemcitabine is an antimetabolic drug. It is an antagonist of pyrimidines and competes with the physiological nucleotide deoxycytidine during DNA synthesis. Gemcitabine exerts antitumor activity in pancreatic cancer, non-small cell lung cancer, breast cancer, bladder cancer, ovarian cancer, and sarcoma *via* intracellular activation and degradation (Gandhi and Plunkett, 1990; Sandler et al., 2000; Von Der Maase et al., 2000; Albain et al., 2008).

Data from mouse models of colon cancer suggest that resistance to Gemcitabine may be the result of increased metabolic degradation of Gemcitabine into difluorodeoxyuridine by the long isoform of the bacterial enzyme cytidine deaminase (CDDL), mainly found in the γ-amastigotes phylum (Geller et al., 2017; Choy et al., 2018). Indeed, tail vein injection of cdd wild-type *E. coli* but not cdd-deficient *E. coli* was associated with Gemcitabine resistance in mice with subcutaneous colon cancer induced by MC-26 cells. This resistance was caused by increased degradation of Gemcitabine by bacterial CDD. In contrast, in the presence of cdd-deficient *E. coli*, Gemcitabine was not degraded to inactive metabolites and thus could inhibit tumor growth. Furthermore, Ciprofloxacin could enhance the antitumor activity of Gemcitabine by inhibiting the growth of bacteria. These results also suggest that gut microbiota can affect the antitumor activity of Gemcitabine in mouse models.

In addition to gut microbiota, the bacteria in the tumor environment can also affect the response to cancer therapy. They can alter the chemical structure of chemotherapeutic agents, and affect their activity and local concentration (Lehouritis et al., 2015; Panebianco et al., 2018b). Geller et al. (2017) analyzed tissue samples from the normal human pancreas and pancreatic cancer. They found that bacterial DNA was found in 86 (76%) of 114 human pancreatic tumor samples, while bacterial DNA was found in only 3 (15%) of 20 normal human pancreatic samples. The most common species in human pancreatic tumor samples was *γ-amastigotes*. The *phylum Amastigotes* is abundant in the duodenum, and the bacteria in the pancreatic tumor samples may be from the duodenum through retrograde migration. In addition, the immune cells in the tumor microenvironment may be suppressed, resulting in the residence of bacteria (Geller et al., 2017). In a mouse colon cancer model, *γ-amastigotes* were shown to trigger Gemcitabine resistance, which could be antagonized by Ciprofloxacin. These results suggest that there may be bacteria in human pancreatic ductal adenocarcinoma that may modulate the antitumor activity of Gemcitabine (Choy et al., 2018).

Other studies have confirmed *in vivo* that *E. coli* can impair the efficacy of Gemcitabine (Lehouritis et al., 2015). Thus, treatment of cancer synergistically with Gemcitabine and antibacterial drugs may help improve the treatment efficacy.

### Platinum

Platinum-based antitumor drugs, such as Oxaliplatin and cisplatin, can target DNA. They can not only induce the formation of platinum-DNA adducts/crosslinks, which block DNA replication and the production of reactive oxygen species (ROS), but also stimulate the immune response (Panebianco et al., 2018b; Lin et al., 2019; Wong et al., 2019). Their efficacy depends on the microbiome and the immune system.

#### Cisplatin

Cisplatin, either alone or in combination, is widely used for the treatment of several advanced solid tumors, such as the head and neck cancer, ovary cancer, cervical cancer, biliary tract cancer, lung cancer, and testicular cancer. Cisplatin is known to exert antibiotic effects on both Gram-negative and Gram-positive strains, such as *Bacillus* and *E. coli* (Joyce et al., 2010).

Gut microbiota also seems to affect the anticancer activity of cisplatin. Reduced anticancer efficacy of platinum salts, in fact, has been reported in animals treated with anti-Gram-positive antibiotics. These effects are associated with the translocation of Gram-positive bacteria during mucositis and subsequent induction of cytotoxic ROS and tumor infiltration by pathogenic Th-17 cells. Indeed, Pflug et al. (2016) reported a potential negative effect of antibiotics against Gram-positive bacteria on the anticancer activity of cisplatin (Iida et al., 2013). Iida et al. found that probiotics such as *Lactobacillus acidophillus* could stimulate the secretion of ROS from immune cells, which could enhance DNA damage, block DNA repair and transcription, and lead to cell death, thereby enhancing the efficacy of Platinum; while, the secretion of ROS decreased when mice were deficient in intestinal probiotics. Similarly, in antibiotic-treated mice, probiotics such as *Lactobacillus acidophillus* could restore the antitumor effects of cisplatin (Iida et al., 2013).

#### Oxaliplatin

Oxaliplatin is a 3rd generation platinum anticancer drug and a platinum analogue of diaminocyclohexane, in which the amino group of cisplatin is replaced by a 1,2-diaminocyclohexane group. Its mechanism of action is to target DNA and inhibit DNA replication, which is similar to that of other platinum analogues. It has synergistic effect in combination with 5-Fu. *In vitro* and *in vivo* studies have shown no cross-resistance with cisplatin, widely used in the treatment of gastrointestinal tract tumors.

The tumor-inhibiting effect of oxaliplatin depends on the microbiome. The efficacy of oxaliplatin is decreased due to the reduced production of ROS in germ-free mice (Iida et al., 2013). In addition, when antibiotics are used, the recruitment of immune cells important for mediating tumor suppression is reduced, and their pro-inflammatory potential is also reduced. This finding suggests that the microbiome exerts immunomodulatory effects in response to chemotherapeutic agents (Wilkinson et al., 2018).

Gut microbiota (Garrett, 2015; Rea et al., 2018) stimulates the production of ROS in tumor-infiltrating myeloid cells. High levels of ROS lead to oxidative stress, which results in Oxaliplatin genotoxicity and triggers cancer cell death (Lin et al., 2019). It is suggested that the TLR4-MYD88 signaling pathway is involved in this process (Cogdill et al., 2018; Von Frieling et al., 2018). Iida et al. (2013) showed that Oxaliplatin treatment induced a reduction in tumor volume and survival rate in antibiotic-treated mice when compared to normal mice. The possible mechanism is that the efficacy of Oxaliplatin depends on the activation of myeloid cells by the gut microbiota, which releases ROS to stimulate Oxaliplatin activity. Disruption of the gut microbiota homeostasis reduces the production of ROS, leading to reduced efficacy of chemotherapy.

The disruption of gut microbiota composition can reduce the efficacy of platinum drugs, suggesting that the microbiome plays a key role in regulating the efficacy and toxicity of chemotherapeutic drugs (Wilkinson et al., 2018). It is reported that in mouse models without reproductive/antibiotic treatment, there was a decrease in microbial-induced ROS production, leading to the failure of chemotherapy (Von Frieling et al., 2018). However, lipopolysaccharide administration (Wilkinson et al., 2018) rescued the chemotherapy failure. In addition, Oxaliplatin induces translocation of specific bacterial species, such as *Lactobacillus yohimbe* and *Enterococcus hirsutus*, from the intestinal lumen into the secondary lymphoid organs, which leads to the initiation of a Th1 memory response. Interestingly, it is shown that other chemotherapy drugs, such as alkylating agents, anthracyclines, bavodotoxins, chymotrypsin, and posterior toxins, can exert similar effects as platinum-based chemotherapy drugs by producing high levels of ROS (Lin et al., 2019). These data highlight the improving effects of gut microbiota on the efficacy of cancer chemotherapy (Iida et al., 2013; Perez-Chanona and Trinchieri, 2016).

## Effect of gut microbiota on adverse effects of chemotherapeutic drugs

### 5-Fu

In addition to acquired resistance, the clinical application of 5-Fu is limited by gastrointestinal toxicity and mucositis. Understanding the impact of gut microbes on 5-Fu-related toxicity may help identify potentially targets (i.e., the bacteria themselves or bacterially mediated pathways), thus reducing the side effects of chemotherapy.

Through metabolism such as hepatic and enterohepatic circulation, chemotherapy drugs can affect the homeostasis of gut microbiota. 5-Fu can lead to an imbalance of gut microorganisms. Even a single intraperitoneal injection of 5-Fu (Lin et al., 2019) can induce mucositis throughout the gastrointestinal tract from the mouth to the anus, and make the mucosal tissues prone to ulceration and infection (Pereira et al., 2016). The subsequent inflammation leads to increased gut mucositis and may result in bacteremia and sepsis. After 5-Fu application, the relative abundance of parthenogenic Gram-negative bacillus in the oral cavity and gut microbiota of rats were increased, and mucosal barrier function was impaired. The number of gut bacteria translocated to the cervical and mesenteric lymph nodes were increased. Several preclinical studies have reported a dramatic shift from commensal bacteria (i.e., *Bifidobacterium* and *Lactobacillus*) to *E. coli*, *Clostridium*, and *Enterococcus* spp. (Nijhuis et al., 2017). The formation of ROS and the production of pro-inflammatory cytokines, such as IL-1β, IL-6, and tumor necrosis factor-α, are reported to be involved in 5-Fu-induced mucositis (Pereira et al., 2016).

However, the pathogenesis of mucositis induced by 5-Fu is not fully elucidated by the preclinical studies. There is growing evidence that gut microbiota may play a role in 5-Fu-induced mucositis. Yuan et al. (2018) investigated gut microbiota in 5-Fu treated mice. By using a mouse model of colorectal cancer and high-throughput sequencing, they demonstrated that the antibiotic highly active antiretroviral therapy reduced the antitumor efficacy of 5-Fu in mice. However, supplementation of probiotics after 5-Fu treatment did not significantly increase the efficacy of 5-Fu treatment.

### Cisplatin

Cisplatin may bind to DNA and thereby affect DNA replication in rapidly proliferating epithelial cells, which in turn leads to loss of gut mucosal integrity. This damage can disrupt the mucosal barrier, and result in the development of potentially life-threatening infections (Taur and Pamer, 2016). Reconstitution of gut bacteria altered by cisplatin accelerates gut epithelial healing and improves systemic inflammation. Thus, fecal microbiome transplantation may potentially prevent life-threatening sepsis in cancer patients under treatment with cisplatin (Perales-Puchalt et al., 2018).

Gut microbiota is involved in regulating other common side effects of cisplatin, such as ototoxicity, mucositis, and weight loss. Campbell et al. (1996) that D-methionine had a protective effect against cisplatin-induced ototoxicity in rats. A double-blind placebo-controlled multicenter phase II trial (Hamstra et al., 2010, 2018) showed that oral D-methionine could protect against cisplatin-induced mucositis but did not affect the tumor response to cisplatin. Wu et al. demonstrated (Wu et al., 2019) that D-methionine not only protected against cisplatin toxicity through its antioxidant and anti-inflammatory properties but also regulated cisplatin-induced gut microbiota imbalance by promoting the growth of beneficial bacteria (*Lachnospiraceae* and *Lactobacillus*). Besides, another study also suggested (Zhao et al., 2018) that alterations in the gut microbiota, particularly the decrease in the thick-walled phylum and *Lactobacillus*, may be the mechanism responsible for the side effects of weight loss and cardiac dysfunction induced by cisplatin. Oral supplementation with lactobacilli can prevent weight loss and restore heart function (Ding et al., 2018).

### Irinotecan

As an inhibitor of topoisomerase I and an S-phase cell cycle-specific antitumor agent, irinotecan has been used in combination with other anticancer drugs for the treatment of advanced colorectal cancer, pancreatic cancer, and small cell lung cancer (Ding et al., 2018). Diarrhea is its dose-limiting toxicity (Alexander et al., 2017; Wilkinson et al., 2018). Its activation of SN-38 metabolite after clearance by carboxylesterases is followed by glucuronidation to the inactive form of SN-38G in the liver, which is eliminated by biliary excretion. Once in the gut, SN-38G is reduced by bacterial β-glucuronidase into SN-38, which can cause immediate gut damage and diarrhea. This type of diarrhea is usually treated with loperamide. The toxicity of SN-38 is due to damage to crypt cells in the cecum, as well as the induction of submucosal inflammation (Wilkinson et al., 2018).

Irinotecan itself may enhance its toxicity by increasing the colonization of β-glucuronidase-producing species such as *E. coli*, *Staphylococcus*, *Clostridium cluster XI*, and *Enterobacteriaceae* in the gut (Takasuna et al., 1996; Stringer et al., 2008; Lin et al., 2014). To prevent dose-limiting diarrhea due to irinotecan treatment, alternative strategies have been developed.

One strategy is to use antibiotics to alleviate treatment-related diarrhea. It has been shown that germ-free mice can receive higher doses of irinotecan and that these mice exhibit less gastrointestinal damage than mice with an intact microbiome (Brandi et al., 2006). Although the use of antibiotics inhibits the cytotoxicity of irinotecan (Wilkinson et al., 2018), it also reduces the number of ß-glucuronidase-producing bacteria. Furthermore, studies on rat models have shown that Amoxapine effectively suppresses the diarrheal symptoms associated with irinotecan (Alexander et al., 2017; Pouncey et al., 2018).

Another alternative approach is to use the bacteria-specific inhibitors. It has been reported that the combined use of irinotecan and selective inhibitors for bacterial β-glucuronidase prevented colonic injury or the development of diarrhea compared to irinotecan alone (Gui et al., 2015). Four potent inhibitors of ß-glucuronidase have been identified (Pollet et al., 2017; Creekmore et al., 2019). *In vitro* or *in vivo*, these inhibitors do not affect bacterial cell growth or survival under aerobic or anaerobic conditions, nor do they kill mammalian epithelial cells. Irinotecan combined with bacterial ß-glucuronidase inhibitors protected mice from irinotecan-associated diarrhea (Wallace et al., 2010). These data support the hypothesis that inhibition of the ß-glucuronidase prevents the gastrointestinal toxicity of irinotecan metabolites.

In a rat model of colon cancer, irinotecan increased the abundance of *Clostridium IV clusters* and *Enterobacteriaceae bacteria*, and these bacteria, which are generally low in healthy humans and rodents, could cause diarrhea (Lin et al., 2012). In addition, a relationship between changes in fecal microbiota and drug-induced gastrointestinal toxicity was revealed in irinotecan-treated rats. The study observed a significant decrease in microbial diversity and an increase in *Fusobacterium* and *Proteus*, both of which were associated with gut inflammation.

## Conclusion and outlook

There is a dynamic balance between symbiosis and pathogenesis in human microbiota, which can affect almost all aspects of host physiological functions. Over the past few decades, the gut and tumor microbiota has become a promising area in cancer therapy. There is growing evidence that gut and tumor microbiota can modulate the host response to chemotherapeutic drugs through a variety of mechanisms, including immunomodulation, heterogeneous metabolism, and altered community structure. However, the mechanism of tumor-microbe-host-drug interactions is still unclear. The high complexity of microecology system of the microbiota remains a major obstacle to understanding its detailed mechanisms. Although challenging, it is worthwhile to improve the efficacy and reduce the side effects of chemotherapeutic drugs *via* modulation of the microbiota. In addition, it is shown that cancer treatment responses can be modulated by gut microbiota, such as probiotics or prefabricated fecal microbiota transplantation. In the future, these approaches can be used to achieve precise modulation of gut microbiota composition, providing insights into personalized chemotherapy regimens and offering opportunity for the development of new treatments. Therefore, for precise tumor treatment, it is of critical value to explore the effects of gut and tumor microbiota on the antitumor efficacy of chemotherapeutic agents, which may provide possible targets for the next generation of cancer therapy.

Although the effects of gut and tumor microbiota on antitumor efficacy and adverse effects of chemotherapy drugs have been widely studied, many issues remain unresolved. First, the mechanism by which the microbiota promotes the immune response remains unclear. The dual role of the gut microbiota on tumors confirms the complexity of the bacteria-immunity-tumor axis. It is unclear which gut microbiome composition can best promote antitumor immune responses, and this needs to be investigated through clinical trials. Most studies are limited to animal models. Due to the differences in the gut microbiota between humans and animals, such as the types, numbers and proportions of microbiota, more clinical data are needed to support the clinical application of these experimental results. Secondly, the anti-tumor efficacy of bacteria has been studied in only few tumor types and drugs, and clinical researches on more types of tumors and drugs are lacking. Further researches are needed to determine the relationship between the two. In addition, the current researches have certain limitations. For example, there is the potential for microbial contamination as a confounder of studies using samples from tumor tissues with a low microbial biomass. However, these issues will surely become the hot spots in the frontiers of cancer precision medicine research in the future. Our ultimate goal is to develop a single type or a combination of gut bacteria that can not only prolong the action time of drugs and promote anti-cancer treatment, but also reduce the adverse effects of drugs and ensure the smooth progression of anti-cancer treatment. Therefore, intervention on the gut microbiota is likely to become another frontier in the treatment of malignant tumors.

## Data availability statement

The data that support the findings of this study are available from the corresponding author upon reasonable request.

## Author contributions

YL and PL designed the study. BY, XW, and FY collected the data. BY wrote the paper. PL collected the fund. YL and PL revised the paper. All authors have rad and approved the paper.

## Funding

This work was supported by the Major Science and Technology Innovation Project of Shandong Province [2018CXGC1220] and Shandong Medical and Health Science and Technology Development Project[202003101013].

## Conflict of interest

The authors declare that the research was conducted in the absence of any commercial or financial relationships that could be construed as a potential conflict of interest.

## Publisher’s note

All claims expressed in this article are solely those of the authors and do not necessarily represent those of their affiliated organizations, or those of the publisher, the editors and the reviewers. Any product that may be evaluated in this article, or claim that may be made by its manufacturer, is not guaranteed or endorsed by the publisher.
